# *“I’m not being rude, I’d want somebody normal*”: Adolescents’ Perception of their Peers with Tourette’s Syndrome: an Exploratory Study

**DOI:** 10.1007/s10882-016-9524-y

**Published:** 2016-11-18

**Authors:** Melina Aikaterini Malli, Rachel Forrester-Jones

**Affiliations:** Tizard Centre, University of Kent, Canterbury, CT2 7NF UK

**Keywords:** Tourette’s syndrome, Peer relationships, Grounded theory, Downward social comparison, Attribution theory, Benevolence stigma

## Abstract

Tourette’s syndrome (TS) is a highly stigmatised condition, and typically developing adolescents’ motives and reasons for excluding individuals with TS have not been examined. The aim of the study was to understand how TS is conceptualised by adolescents and explore how individuals with TS are perceived by their typically developing peers. Free text writing and focus groups were used to elicit the views of twenty-two year ten students from a secondary school in South East England. Grounded theory was used to develop an analytical framework. Participants’ understanding about the condition was construed from misconceptions, unfamiliarity and unanswered questions. Adolescents who conceived TS as a condition beyond the individual’s control perceived their peers as being deprived of agency and strength and as straying from the boundaries of normalcy. People with TS were viewed as individuals deserving pity, and in need of support. Although participants maintained they had feelings of social politeness towards those with TS, they would avoid initiating meaningful social relationships with them due to fear of ‘social contamination’. Intergroup anxiety would also inhibit a close degree of social contact. Participants that viewed those with TS as responsible for their condition expressed a plenary desire for social distance. However, these behavioural intentions were not limited to adolescents that elicited inferences of responsibility to people with TS, indicating that attributional models of stigmatisation may be of secondary importance in the case of TS. Implications for interventions to improve school belonging among youth with TS are discussed.

## Introduction

Tourette’s syndrome (TS) is a complex, hereditary neurodevelopmental disorder with a childhood onset. Its main characteristics are tics; non-rhythmic, non-purposeful movements and vocalisations that change over time and fluctuate in severity, frequency and intensity. They are typically associated with irresistible urges and are suggestible and suppressible. TS is clinically typified by the presence of multiple motor and at least one vocal tic for a period of no less than a year (American Psychiatric Association 2013). Onset typically occurs before the age of 18 years, with boys three times more likely than girls to be effected (Bloch and Leckman 2009). The tics vary in complexity from simple eye blinks and coughing to more complex gestures and vocalisations such as touching objects and repeating parts of words and phrases. The severity of the tics vary widely between individuals. The symptomology follows a remitting course and less than one fourth of individuals with TS report moderate or severe tic symptoms in adulthood (Bloch 2013). Although once considered to be a rarity and underestimated due to its complex clinical condition, recent epidemiological international studies suggest that it can be encountered in approximately 1 % of the school aged population (Knight et al. 2012). This rate is consistent with UK studies of school children (Stern et al. 2005) as well as the overall global prevalence rate of adults reported (Robertson 2008; Robertson et al. 2009). Nevertheless, caution must be exercised when reporting cross-cultural prevalence of TS since different countries tend to use different assessment methods (self-assessment, clinician assessment etc). There also appears to be lower prevalence rates among Asian and South African populations though again, this might be due to different criteria for diagnosis (Robertson 2008).

According to both clinical and community studies the disorder is also associated with increased prevalence rates of co-occurring disorders (90 %), the most common ones being Attention Deficit Hyperactivity Disorder (ADHD) and Obsessive Compulsive Disorder (OCD) (Freeman et al. 2000). Increased tic severity and the presence of comorbidities is associated with social difficulties and diminished Quality of Life (QoL) with strong adverse effects in the social domain (Storch et al. 2007).

There is a growing body of research that demonstrates individuals with Tourette’s experience social difficulties and endure social stigmatisation (Malli et al. 2016; Smith et al. 2015). A recent systematic review and synthesis indicated that children and adolescents with Tourette’s syndrome report impaired peer relations and are at a significantly higher risk of stigmatisation and victimisation compared to their typically developing peers, including name-calling, relational bullying, teasing, ridicule, discrimination and marginalisation (Malli et al. 2016).

It has been suggested that individuals with TS may experience difficulties due to poor social functioning (Güler et al. 2015) and lower levels of social competence in comparison to normative peer groups (Zhu et al. 2006). Deficits in social and adaptive functioning may be further exacerbated by co-occurring conditions such as ADHD (Carter et al. 2000) and anxiety disorder (Gorman et al. 2010).

Individuals with TS may also experience social maladjustment due to the low social acceptability of the tics. The syndrome has been characterised as an “illness of the observer” since the most catastrophic outcomes of the tics occur in the spectator’s mind (Hollenbeck 2003). Indeed, a cluster of studies within controlled settings extending across gender and age have demonstrated that individuals with TS are perceived as less socially acceptable than their tic-free peers (Boudjouk et al. 2000; Friedrich et al. 1996; Holtz and Tessman 2007; Woods et al. 1999; Woods 2002; Woods and Marcks 2005). Specifically, Friedrich’s research indicated that typically developing children rated a peer with tics as a less desirable playmate. These findings were further replicated by Woods’s studies which demonstrated the negative social effects of tics within cohorts of college students. It is therefore possible that children and adults with TS are confronted with negative attitudes and are at risk of social rejection due to their tics.

One of the limitations of the literature to date is that considerably less attention has been given to understanding adolescents’ awareness and attitudes towards their peers with TS. Thus, research has been primarily directed towards young children and college-age youth. Although the intensity and frequency of the tics usually decline during puberty, according to the clinical perspective, adolescence is the period in which individuals with TS experience their symptoms as most challenging and when exclusionary practices become more prominent (Sukhodolsky et al. 2013). Due to the negative evaluation and stigma associated with TS, individuals with the condition may have limited chances to develop social adaptive skills and supportive social relationships, both poignant developmental challenges for adolescence (Sullivan 2013). Therefore the focus of research should be placed on the specific age-group.

Another limitation relating to previous studies of attitudes towards Tourette’s syndrome is that they have been predominantly based on narrow questionnaires that solely investigate social acceptability and overt prejudice and not social participation and friendships. Furthermore, possible explanations and motives about the causation of social exclusion have not been provided. The aforementioned measures have also failed to uncover underlying biases that are more likely to occur in today’s society where overt discrimination is considered morally reprehensible and the tendency to supress inappropriate forms of prejudice is more pronounced (Bergsieker et al. 2012). Finally, only a limited number of studies have examined the knowledge base typically developing peers have about TS and the possible impact that this knowledge might have on their subsequent behaviour. Therefore, a nuanced and substantive understanding of how typically developing adolescents perceive their peers with TS that discerns their views on forming relationships with them is lacking in the literature.

Interestingly, studies to date suggest that stigma towards individuals with TS is robust and resilient to change and there have been a number of unsuccessful interventions (Friedrich et al. 1996; Woods and Marcks 2005). Therefore, more interventions need to be developed that are not solely based on theoretical principles but explicitly address the concerns of specific subgroups. A theory about typically developing adolescents’ perception towards peers with Tourette’s syndrome could help identify modifiable causal factors associated with attitudes and support the development of more tailor-made interventions in order to positively change the referent age-groups’ knowledge, attitudes and behavioural intentions.

In an attempt to address the gaps in the literature the current study aimed to explore how adolescents with TS are perceived by their peers from a qualitative stance in order to provide a richer understanding of the phenomenon. More specifically the study aims were as follows:

1. To investigate typically developing adolescents’ knowledge about TS, expressed in their own words.
2. To explore the perception and feelings of tic-free adolescents towards individuals who do have TS utilizing a range of qualitative methods.
3. To assess typical students’ intent to interact with individuals with TS and to provide a comprehensive understanding of their behavioural intentions.

The overall objective of the study was to gain insight into how the condition is conceptualised by adolescents who do not have Tourette’s syndrome, and to provide further data on this under-studied subject of how adolescents with TS are perceived by their typically developing peers.

### Setting and Sample

Participants were adolescents in a co-educational, multi-cultural comprehensive secondary school in South East England. The pupils were of low attainment and more than one quarter were recorded as having English as an Additional Language (EAL). There were 33 languages spoken altogether including Polish, Czeska, and Lithuanian with Slovak and Latvian being the most common first languages.

The study’s inclusion criteria stipulated that the participants had to be English speaking pupils between the ages of fourteen to seventeen. Although English did not have to be their first language it was imperative for the study that they could speak English fluently, in order to provide meaningful qualitative data. Due to the fact that the school had so many pupils with limited English skills, the participants for the study were handpicked from various classes in Year 10 (14–15 years old).

Participants included twenty two year-10 pupils (17 females and 5 males). Regarding the participants’ first language 81.8 % reported it being English, 4.5 % Latvian, 4.5 % Slovak, 4.5 % Turkish and 4.5 % Russian. Two participants (9.1 %) reported having a relative with TS, half of the sample had previously watched a show on TV that included a person with Tourette’s syndrome, and 9 (40.9 %) had never observed a person with Tourette’s syndrome.

### Data Collection

Focus groups and free text writing were combined to achieve a nuanced and enhanced understanding of the social phenomenon under investigation. Free text writing provided a concrete individual-level data set that was not skewed from or influenced by the norm of the peer group (Murphy and Dingwall 2003). On the other hand, focus groups provided the chance to elicit the shared understanding and meaning of the social phenomenon (Kitzinger 1994). Thus, by combining attitudes expressed privately, with those articulated within group discussions a more comprehensive understanding of the structure of the phenomenon was more likely to emerge (Lambert and Loiselle 2008).

### Free Text Writing

All participants were given ten minutes to express their thoughts and feelings in relation to individuals with TS through free text writing. Instructions were written as follows:

“We want to know what you think about people with Tourette’s syndrome. Please remember that no one else apart from the researchers, will see what you have written about, so feel free to express your thoughts and feelings.

What do you think about people with Tourette’s syndrome? How do they make you feel?”

Free text responses were deemed appropriate for the current research since this method can elicit rich data by providing the research participants with the opportunity to express their thoughts in their own words, using their own vocabulary. Participants were encouraged to write as much or as little as they wished; express themselves in their own words; and were also discouraged from evaluating the quality of their text in terms of writing skills such as spelling, punctuation and neatness so as not to detract from the focus of thinking about TS. Similar to questionnaires free text writing can also elicit thoughts that participants might not be willing to disclose in a face-to face interview. Responses may also identify issues and perspectives that might not have been covered in more forced choice questionnaires (Garcia et al. 2004).

### Focus Groups

Each focus group consisted of four to six participants. These clusters were arranged by the teacher based on close friendship networks between the participants. It has been argued that discussions conducted between members of a friendship group hold very close resemblance to naturally occurring dialogues (Kitzinger 1994). These settings might also give some “hesitant” interviewees the impetus to participate (Puchta and Potter 2004). In contrast to individual interviews youths are more likely to disclose personal thoughts and attitudes within a group of peers than they are to an adult interviewer to whom they most probably will express opinions and views that comply with the social norm (Krumpal 2013).

The interview guide was developed from gaps about attitudes towards individuals with TS identified through the literature review. Data from the free text writing also determined pertinent questions that were further explored in the group context.

During the focus groups participants were also introduced to two vignettes in which the protagonist, a typically developing adolescent, was faced with a moral dilemma about the social inclusion or exclusion of a peer with TS in two different social contexts. The use of the vignettes was twofold: i) to elicit the participants’ judgement regarding the protagonists’ decision and ii) to facilitate discussion about the participants’ moral reasoning when making their decisions about peer group exclusion and inclusion (Horn et al. 1999). The intention of the first story was to understand if rejection is considered permissible because an individual with TS does not assimilate to the norms of a group. The second vignette was utilised in order to assess if exclusion was justified as a result of personal choice.

### Procedure

The study was reviewed and given an ethically favourable opinion by the University of Kent Ethics Committee (January 2015). Information sheets explaining the study were provided for both parents and potential participants. Opt-out consent forms were given to parents and assent forms to the participants were distributed by the school before the start of the study. No one refused to participate.

A pilot with four students outside of the study sample was conducted one week before the main study and since no significant changes were made to the research instruments the pilot data was included in the study’s main results. This is common practice in qualitative studies since consecutive interviews help the researcher progressively gain more insight and focus in the interview schedule (Cutler et al. 2009; Holloway 1997).

The study was undertaken during the students’ regular school timetable. Participants were gathered into a large school room during their regular 40 min classroom period. First a four minute video was used to introduce the participants to the symptomology of Tourette’s syndrome. The video which was obtained from the public domain depicted a Caucasian adolescent girl, of approximately the same age as the participants, who exhibited TS symptomology, including simple and complex tics both motor and phonic in the form of repetitive eye-blinking throat-clearing sounds, grunting, sniffing, coughing, head jerking, neck stretching and facial grimacing. The individual was viewed from the waist up while sitting in a chair in front of a desk that was against a neutral background. The girl briefly introduced herself and mentioned that the purpose of creating the video was to demonstrate the transient exacerbation of tics while she was engaged with homework (see https://www.youtube.com/watch?v=O6j8YM1CcPU). The accurateness of the symptomology displayed was assessed by four parents of children with TS who belonged to a TS support association outside the main study and its validity was also academically peer reviewed for the purpose of this study. Each of the groups concurred that it accurately reflected TS behaviour. This video overcame the limitations of the videos used in previous studies (Boudjouk et al. 2000; Friedrich et al. 1996) in which only motor tics were exhibited as the basic component of TS. Furthermore, by using a video of an actual individual with TS as opposed to an actor simulating tics, the external validity of the study was enhanced.

After viewing the short stimulus video participants were asked to complete a demographic and free text writing measure. The pupils were closely monitored by the researcher and a school teacher in order to prevent any sharing of information in regards to their responses and to ensure that all of the measures had been filled in. During the following week the focus groups were facilitated by the first author during three consecutive 40 min classroom periods within the school premises. A school staff member was sitting at the back of the room in each of the sessions.

### Data Analysis

The data obtained from the free-text writing and focus group were collated in order to identify a comprehensive model for the phenomenon under study. Grounded theory (Glaser and Strauss 1967; Strauss and Corbin 1998) guided the data analysis which was deemed appropriate due to its suitability to explore subjects that have been relatively under studied (Goulding 2002). Furthermore, it was the intention of the study to move beyond a thick description of the social phenomenon to the development of an explanatory theoretical model grounded in the data.

The transcripts and free text writing were initially rigorously read multiple times by both authors who independently coded and categorised the data. Patterns of categories were subjected to a process of ‘constant comparison’ between the codes and the categories in order to adjust and refine them. Categories incorporated several codes under a more conceptual heading. During the next stage, the categories were explored further and higher order categories (or sub-themes) were identified that moved beyond description to interpretation. Elongated discussions between the authors comparing categories and sub-themes followed until saturation and also to ensure thematic-reliability. This process yielded one overarching theme, three sub-themes and seven categories.

**Fig. 1:**
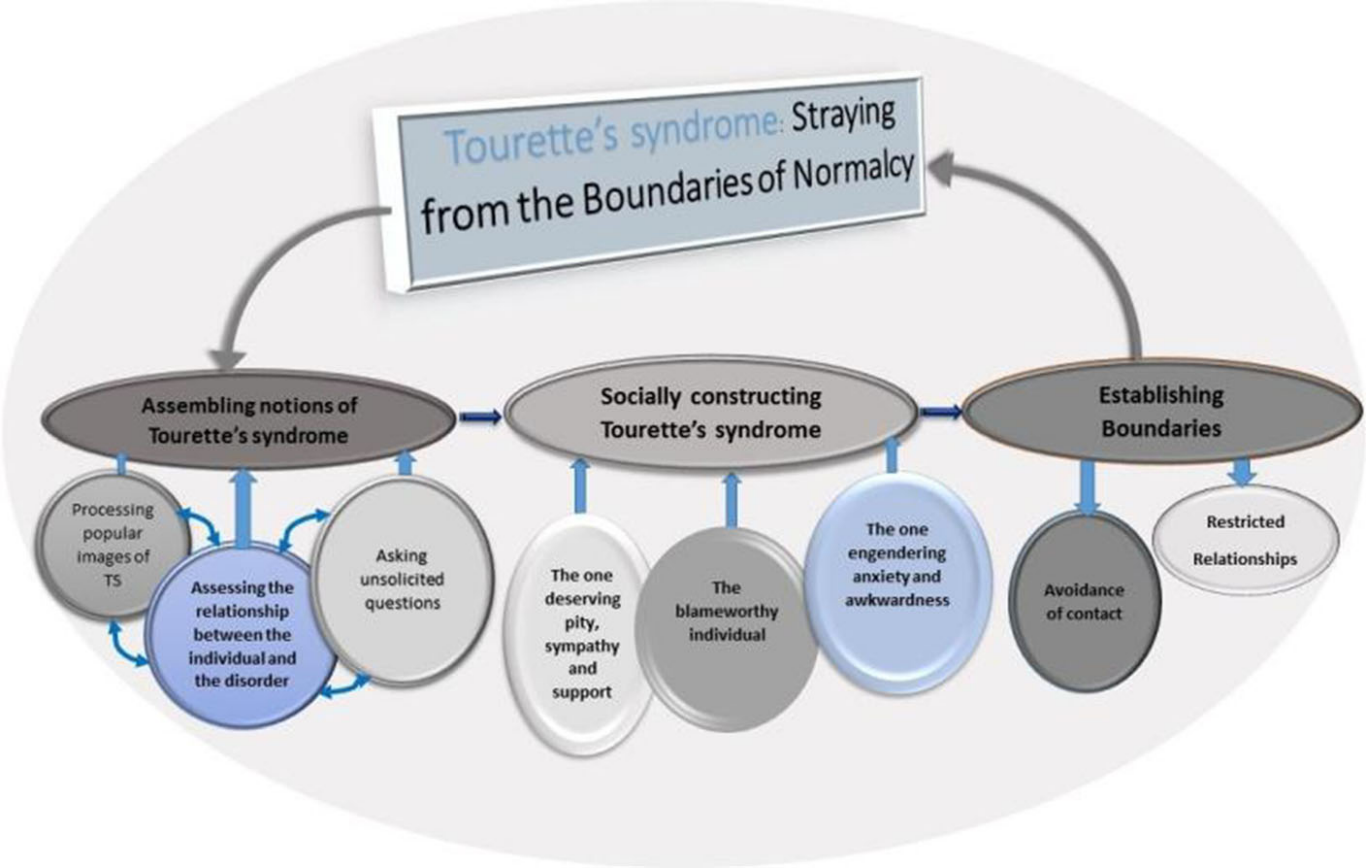
Process by which typically developing adolescents perceived their peers with Tourette’s syndrome

Causal conditions, contexts, and behavioural outcomes relating to the studied phenomenon were uncovered in order to identify a model of how individuals with TS are perceived by their typically developing peers. Memos pertaining to hypotheses about the relationship between the categories and sub-themes were used to facilitate a more theoretical understanding of the data. This was aided by diagrammatic representations of possible relationships between the categories and sub-themes constructed in a systematic and rigorous way (Fig. 1). NVivo 10, a software tool for managing qualitative research data was used to facilitate the grounded theory approach (Hutchison et al. 2010).

## Results

The overarching theme to emerge from the data was ‘Straying from the boundaries of normalcy’ as illustrated in Fig. 1. The figure demonstrates a conceptual framework for understanding the process by which the participants cognitively conceptualise Tourette’s syndrome, their subsequent affective responses and their behavioural intentions towards their peers with TS. Three key sub-themes were identified as being involved in the process: ‘Assembling notions of Tourette’s syndrome’; ‘Socially constructing TS’; and ‘Establishing boundaries’. Participants appeared to construct their notion about TS by processing popular cultural images of TS, assessing the responsibility the individual has over the condition and asking questions about the syndrome. This social construction of TS led to a perceived social identity which the adolescents projected onto a person with TS. Most participants identified individuals with TS as deserving pity and sympathy whilst others viewed them as responsible for their condition. Individuals with TS were also perceived as people that would make typically developing adolescents feel anxious and awkward. This socially constructed social identity of people with TS which was bound up in pejorative ideas about the condition defined the subsequent behaviour of the participants towards their peers with TS; establishing boundaries by either avoiding contact or creating restricted relationships with their peers with TS.

The themes and subthemes are described below accompanied with substantive anonymous quotes from participants which have been unaltered by the authors to maintain participant authenticity.

### Assembling Notions of Tourette’s Syndrome

The participants appeared to have largely constructed their own perceptions about the nature of TS through processing images that had been presented in popular culture. In turn, the adolescents made assumptions about the controllability and responsibility individuals have over their symptomology. Adolescents also expressed a keen interest in understanding the condition and familiarising themselves with individuals with TS, indicating that the amount of information they had previously received had been insufficient.

### Processing Popular Images of Tourette’s Syndrome

Participants reported that they had experienced relatively little contact with individuals with TS although the majority were aware of the syndrome through images on the TV and the internet. Erroneous beliefs prevailed among them pertaining to the core feature of the condition. Both documentaries and movies that the participants recalled focused on coprolalia, the uncontrollable utterance of obscenities and profanities (American Psychiatric Association 2013). In the movies they mentioned such as “Not Another Teen Movie” and documentaries series such as the “Undatables” individuals with TS were reduced to stereotypes for the sake of comedy or, according to the adolescents ‘losers’ who needed help. Conversely, other relevant aspects of the syndrome were omitted. This is exemplified in the following quotes:

*PL3: I didn’t know that Tourette’s also moved your body, I thought it was only swearing but now I take it as a serious syndrome.* (Free text writing)

*TP3:* [Talking about Not Another Teen movie] *Can’t remember what it was called but there was like this cheerleader and she'd get mad and shout out these swear words* (Focus group 3)*.*

The above quotes show how participants were exposed to a media image of TS in which individuals with the condition were rendered as more different than similar to typically developing individuals. By viewing the most extreme cases, TS was conceptualised as a cursing disease that perpetuated the notion of social misfit and ‘otherness’. Indeed, uncontrollable swearing was identified as the main barrier that segregated individuals with TS, impeded social interaction and ultimately distinguished them from their peers to the extent of ghettoizing individuals as indicated below during a discussion about dating:

TP4:…*So it might be easier for them [people with TS] to date somebody with TS*. (Focus group 3)

### Assessing the Relationship between the Individual and the Disorder

The vast majority of the participants reported that individuals with TS had no control over their symptomology and were therefore not responsible for their condition. This highlights the importance of inferences of personal responsibility to a condition in determining the observer’s understanding and emotional response to it (Weiner 1986). The tics were perceived to stem from within the body and people with TS were unable to exert control over their physical movements and discourse:

*TP12: They can't control it, because this comes from inside the body. (Focus Group 2)*

*TP7: They're not really to blame. It’s just like something happens to them and they can't get rid of it. (Focus Group 4)*

Simultaneously, it was implied that Tourette’s syndrome was something external to the autonomous self that violated the person’s agency. Thus, the person’s involuntary actions were imposed on them by the omnipresent syndrome and the real individual was trapped in an uncontrollable body. The profoundly disempowered real self was dominated by the “illness” that prevailed over the passive physical and social agency of the individual:

*TP6: They can't go anywhere without having tics, they can't control it like that. (Focus group 4)*

*TP14: It’s like it forces or something, forces her to react like that (Free text writing)*

A deprivation of agency implies weakness, immaturity or even danger. Indeed, some participants maintained that the uncontrollability and randomness of the tics may inevitably create danger. This led to a feeling that physical distance had to be maintained in the interest of self-preservation.

*TP2: And it would be like scary ‘cause you don't know what’s gonna happen. I will be wondering what they are going to be doing next. (*Focus group 3)

Only a few of the respondents were sceptical about the uncontrollability of the symptomology; insinuating that the tics were exaggerated. They implied that the symptoms of TS could be alleviated if the actors put enough effort and concentration into the specific task:

*PL3: I feel like they're putting it on (Focus group 1)*

*TP14: it looks like you can control it, if like you put like effort, but I don't know (Focus group 4)*

### Asking Unsolicited Questions

Participants expressed a desire to understand the complex nature of Tourette’s syndrome and the subsequent effects it may have on the inflicted individual. Although the participants’ interest in Tourette’s may have merely been sparked by the study itself and therefore have reflected the Hawthorne effect (McCambridge et al. 2014), these queries were initiated by the young people who took part in the study unprompted by the researcher or their teacher. Questions were posed pertaining to the aetiological causes of TS, the genetic predisposition associated to the disorder, the environmental factors that might influence the tics, the course of syndrome and more specifically if the symptomology of the disorder diminishes with age. Adolescents were also curious about the supressibility of the tics and the level of the individual’s control over them. They also expressed an interest in finding out the coping mechanisms of individuals with TS. This set of questions might suggest that despite the participants’ exposure to the exaggerated media portrayals of TS they still have unanswered questions and a desire to familiarise themselves with pertinent information and individuals with TS as indicated below:

*PL3: Is Tourette's genetic? (Focus group 1)*

*TP15: I would like to know if people are born with it or can it be genetic and they get it later on in life? (Free text writing)*

*TP3: I would like to know how she copes with it and keeps herself calm through it? (Free text writing)*

*TP6: Can you control it to a certain extent? Was you born with it? Will it go? (Free text writing)*

### Socially Constructing Tourette’s Syndrome

Based on the limited information about TS available to the pupils and the affective reaction towards the TS population, the adolescents in the study attempted to socially categorise individuals with TS (Turner et al. 1987). The social identity of individuals with TS was socially constructed through a process of social comparison to what the participants understood as “normal”. The perceived differentiating characteristics between the participants and their peers with TS were accentuated and professed as important and salient. Through this procedure people with TS were viewed as ‘others’ and perceived as ‘out-group’ members that possessed characteristics that the adolescents in the study acknowledged as derogatory. Most participants were conflicted between viewing people with TS as a personification of their deficits and subsequently as deserving sympathy and pity and as a source of discomfort. Others perceived them as responsible for their derogation whilst some viewed them as engendering anxiety and awkwardness.

### The One deserving Pity, Compassion and Support

Adolescents in the study compared their perceived own abilities and future opportunities with those of their peers with TS. The latter were viewed as abject and disadvantaged in relation to an ideal of normalcy held by the pupils. This process which can be interpreted within the downward social comparison framework (Finlay and Lyons 2000; Wills 1981) elicited feelings of pity towards individuals with TS and simultaneously enhanced the participants’ own self-esteem. Indeed, participants thoroughly described what they imagined having TS would entail for one’s social standing. A compromised life was delineated in which individuals with TS would endure social isolation, low levels of social connectedness and limited career prospects. Amongst the social difficulties their peers would encounter, teasing and social isolation were the most prominent:

*Moderator: How would you imagine him/her during break or lunch?*

*TP8: Probably alone. Probably one or maybe two friends.*

*TP3:…..Maybe a few people would make a few jokes* [*within the classroom*]. *But then outside probably they might not sit with her and she'll be quiet lonely. (Focus Group 3)*

*TP14: I felt like kind of sorry ‘cause it must be hard for them to do certain things and have friends and stuff. And it must take a lot more effort than everybody else. ‘Cause you have to try and build a relationship with people and it might be a lot harder because of the tics and stuff*. (Focus group 4)

*TP12: In this situation for them it’s much harder to find a friend some people don’t want to be their friend because they has that illness [sic]* (Free text writing)*.*

Some participants maintained that the tics and the subsequent burden they entail could impede individuals with TS from achieving high academic performances:

*TP3: I felt a little sorry for her cause she was trying to do her homework, so the tics might have been distracting her, so she might not have been able to do her work the best that she could have done. So, it might affect her grades (Focus group 3)*

*PL5: I'd feel sorry for him. Not really sure if they’d drop out of school.*

*Moderator: Why would they drop out of school?*

*PL5: Am just thinking, yeah, could they like concentrate with all the twitches? (Focus group 1)*

A considerable number of participants expressed a concern about the potential barriers individuals with Tourette’s may encounter in the future while pursuing and acquiring a job. Conversations were centred on employment discrimination. The tics and ‘socially inappropriate behaviour’ could restrict and limit their career choices and many participants posited that their ‘otherness’ would prohibit them from pursuing positions that would involve public speaking and face-to-face interaction. It was therefore insinuated that jobs that require less public contact would be more appropriate for them:

*TP6: …… like feels like if he wanted a job* [individual with TS] *to speak in front of lots of people, he wouldn't be able to do it, because he wouldn't be able to control it.*

*TP 5: Yeah. In certain jobs they'll not be able to pursuit their dream ‘cause the condition would hold you back...or from certain parts of it. Like if you're talking in front of groups.* (Focus group 4)

By conceptualising individuals with TS as inferior to themselves, disadvantaged and unaccountable of their misfortune, feelings of pity and sympathy were elicited. Indeed, the phrase “I feel sorry for” or “I feel sad for” was used by eleven of the respondents to describe their emotional response towards an individual with TS.

*TP10: Also it makes me feel sad as they can’t have a normal life as they want, they will always have people looking down at them which could lead them to feel bad about themselves (Free text writing)*

*TP11: This girl makes me feel sorry for her as she is different from other people (Free text writing)*

TP3: *I feel bad for anyone who has Tourette’s. Personally, I would hang around them because it’s not their own liking to have such a syndrome (Free text writing)*

Through social downward comparison (Wills 1981) participants re-evaluated their own social status as well as their groups. They elaborated on positive information about their identity in comparison to the less fortunate group of people with TS and enhanced their self-esteem as seen below:

*TP17: We are lucky that we ain't got Tourette (Free text writing)*

*TP 7: I felt sorry for her, but I also felt glad because I didn't have it (Focus group 4)*

Pity in some cases manifested itself in the form egocentric sadness that did not entail benevolence. Thus, although the participants appeared to feel compassion for the target individual, they were not willing to act in a friendly and approachable manner towards their peers with TS.

*TP12: I think it’s very hard to [sic] that person to live like that. I feel a bit sorry, if somebody repeating the same thing..I wouldn’t [want] that person to be in my class (Free text writing)*

However, a number of participants maintained that they would act in an altruistic manner and help their peers in need. Pity implies that the target individual deserves protection, caring and providing for (Thompson et al. 1980). Some of the participants suggested that they would undertake a custodial role even if it was not being asked of them. This emphasises typically developing adolescents’ effort to express socially appropriate and positive attitudes towards peers with TS but simultaneously indicated their conviction that individuals with TS may need to rely on tic-free components for support.

*Moderator: Do you think that he or she is capable of sorting things out by himself or does he need help?*

*TP8: Cause it kind of like depends on how bad the tics are. ‘Cause if actually* [he] *gets tics and he’s with the group, then yeah he can't really do stuff for himself. (Focus group 3)*

*TP15: You know, people will be like taking the mick out of them. They are not like normal people; they need more help than us (Focus group 2)*

The participants’ self-esteem was also elevated by viewing their actions towards their peers with TS as fair and moral. By providing unsolicited help to their peers and not directly discriminating against them, an action which is perceived as unjust, the participants in turn appeared to obtain a positive self-identity for being helpful:

PL5 characteristically mentioned:

*: …. I wouldn't stay out of it, even though he (the individual that’s being bullied) might get angry at me, I wouldn't. Cause at the end of the day I'd know I've done something that would change people’s lives. Yeah! (Focus Group 1)*

### The Blameworthy Individual

A limited number of participants considered the target individuals responsible for their tics. Inferences of responsibility elicited negative emotions and diminished adolescents’ willingness to provide social support to the hypothetical peer. In fact angry responses were more common in the adolescents who insinuated that the symptomology is high on personal control:

*TP9:... I feel like they can't control it but then it seems a little strange that they can't. I don’t know. They should at least try. The other people try, so why shouldn’t they? Otherwise, just let them be on their own (Focus Group 3)*

This concept could be linked to Weiner’s attribution theory (1986; 1995) that posits that people search for the causation of the behaviours of others. An individual that is perceived to have control over their deviant behaviour and is deemed responsible for their condition may elicit negative responses such as anger from the person they are interacting with. Anger may also influence the observer’s behaviour and higher inferences of responsibility are associated with lower acceptance rates and greater exclusion (Juvonen 1991).

### The One engendering Anxiety and Awkwardness

Throughout the study the participants anticipated that discomfort and awkwardness would stem from a social interaction with an individual with TS. It was suggested that anxiety may originate from the inherently novel situation and the lack of clear rules of conduct as well as the lack of affective readiness on the part of the participants. The unsettling situation in which the normal are uncertain about how to behave around the out-group member has been labelled as ‘interaction pathology’ (Davis 1961) as exemplified by the three participants below:

*TP3: I may be a bit nervous around her because if she does something I wouldn’t know what to do (Free text writing)*

*TP16: Honestly, whilst watching the video clip I feel quiet anxious because I wouldn’t know what to do when I am around someone with Tourette’s syndrome (Free text writing)*

*TP5: It made me feel anxious. Yeah, uneasy (Focus group 4)*

The belief that TS may involve unpredictable behaviour is also a factor that may create an anxiety provoking situation (Corrigan et al. 2005). Some participants maintained that the uncontrollability and randomness of the tics might ostensibly create danger. The tics and the unpredictable way they manifest could appear threatening. The perceived lack of control that people with TS had over their tics appeared anxiety provoking for some of the participants:

*TP12: When I’m seen [sic] person that has Tourette’s syndrome I feel very scared. I know they will not going to do anything but how they acting is scares me [sic] (Free text writing)*

Anxiety may also be invoked from conflicting and incompatible feelings that stem from interacting with an individual that is perceived as an outgroup member (Stephan 2014). Thus, some participants’ evaluations contained both positive and negative dimensions. Well intentioned adolescents were motivated by egalitarian values and were concerned about behaving in a non-stigmatising manner towards an individual who they viewed as disadvantaged. Simultaneously, they acknowledged the potential social cost of affiliating with an individual with TS and voiced their negative attitudes in a concealed manner. In other words, the participants tried to maintain a non-prejudiced self-image while expressing negative attitudes and feelings towards the specific outgroup members. Therefore, attitudinal ambivalence was evoked by inconstant and contradictory reactions to individuals with TS which may have resulted in inter-emotion tension (Katz 1981; Thompson and Zanna 1995). Thus, two dissonant emotions or cognitions towards a social target may evoke anxiety towards the specific individual.

*TP18: They are still people and they still have feelings and the right to have friends, even though I reckon sometimes it would get annoying… it would be extremely distracting and sometimes I may feel uncomfortable*. (Free text writing)

This form of cognitive dissonance may create a discomforting experience which in turn might lead to quite extreme and amplified responses (Bell and Esses 2002). Characteristically, in this study a number of adolescents expressed overly positive intensions towards their peers with TS.

*TP13: I think that I would stay with her forever and really sorry for her. (Free text writing)*

*PL5: I'd try to make them like best friends (Focus Group 1)*

Participants also expressed a concern about the negative social consequences of being closely linked to an individual with TS. More specifically they voiced a fear of “social contamination” (Thornberg 2015), and were apprehensive about other in-group members’ reaction. Some of the participants stated that the social encounters with individuals with TS would preferably take place in settings where the tics were less likely to be conspicuous and where less people would be present. Thus, adolescents wanted to maintain a distance from the hypervisibility imposed on the individual with TS. Public exposure would elicit feelings of discomfort, unwanted attention and embarrassment for all parties involved. More specifically, being directly associated with the person with TS could threaten their social image:

TP11: *I don't know yeah, I wouldn't go to the cinema or the malls...if I had the choice I would like parks. I think it's the best place, ‘cause they would much better if… If you go to the cinema or somewhere the mall you would feel uncomfortable other people around would feel uncomfortable, this person feels. You know what I mean right? In the park it's much better. And not only in the park but somewhere like where less people*. (Focus group 2)

Participants anticipated that the devaluation that stems from the “mark” of TS would spread to the people associated with the individual with TS. As a result they feared that they would also be the subject of curiosity and misunderstanding and subsequently devalued and denigrated by their peers. This phenomenon which Goffman (1963) termed as courtesy-stigma suggests that stigmatisation does not only affect the ‘discredited’ but also impacts those who are associated with the stigmatized individuals. The adolescents in this study acknowledged the social risk of bearing courtesy-stigma and explained the mechanism by which devaluation spreads to other people associated with the target individual. They also expressed scepticism towards the likelihood of the stigmatised individual being benefited or even destigmatised as a function of association with a ‘normal’ individual.

*TP14: Like you stand up for them and they're gonna judge you because you're standing up for them.*

*TP5: Other people don't wanna. So you feel embarrassed to. In fact you get even more judged than who their actually judging (Focus Group 4)*

Some of the participants also feared that by being closely associated to a devalued individual their peers may perceive them as bearing similar derogatory attributes as the stigmatised. In the case of a chosen affiliation, such as a romantic partner, adolescents were concerned that their peers might infer that they too beared an invisible ‘mark’ that would make affiliations with higher status individuals impossible. For instance participant TP12 stated:

*If I had that boyfriend* (with TS) *people gonna judge me. Not me, but they're gonna say she's beautiful but he, he is like, has this syndrome. He's not like* (pause*). What’s wrong with her? You know?*” (Focus group 2)

### Establishing Boundaries

By perceiving adolescents with TS as outgroup members the participants of the study expected that they would prefer to avoid contact with them or they would only be willing to form restricted relationships. Interestingly, social distance would be dependent on the severity and the disruptiveness of the tics. Thus, the concealability of the tics were conceived as the prime means of retaining social acceptability since less visible symptoms of the condition suggest more ambiguous boundaries between ‘them’ and ‘us’. In other words by approaching normalcy and eradicating personal differences marginalisation could be attenuated. These findings confirm that the frequency of the tics have effects on how comfortable the observer might feel while interacting with an individual with TS (Woods et al. 1999). The same concept was highlighted when the participants viewed rehabilitation and cure as a means to overcoming the limitations related to the condition:

*Moderator: Could you potentially date somebody with TS?*

*TP6: It depends.. like you can be with them and after a few years it can get better and they could really stop it. Like stop having Tourette's and just be a normal person (Focus group 4)*

### Avoidance of Contact

A limited number of participants asserted that they would preferably maintain a social and physical distance from their peers with Tourette’s syndrome. Irritated responses and a propensity to segregate individuals with TS were linked to the adolescents that were sceptical about their peers’ inability to exert control over their symptomology. In other words the hypothetical peer with TS was viewed as an individual that consciously and deliberately refused to assimilate to the norm. However, anger and the intention to avoid individuals with TS was not limited to participants who viewed having TS as a form of derogation. Participants’ understanding about the uncontrollability of the syndrome and their sympathy towards their peers did not necessarily positively influence their acceptance or their desire to minimise the social distance between them. Possibly fear, discomfort guilt, and pity may have inhibited them from interacting with individuals with TS:

*TP7: I think she can’t help it and to be honest I wouldnt hang round [sic] with her when it gets bad I would feel awkward but I would feel sorry (Free text writing)*

*PL5: Having a person like that in my class would be very annoying and I would not like to sit next to her because she looks very stupid and childish the things she does and the noises she makes are very annoying and it would be very uncomfortable to sit with her or hang around with her (Free text writing)*

*TP1: So if it's really bad then yeah maybe like he should go like to another school. But umm, but if it's not like that bad, that's fine. Then maybe he should be with everyone else (Focus group 4)*

### Restricted Relationships

Exclusion, in the form of overt rejection was perceived negatively by most of the participants across the four groups. Consistent with Killen’s research (Killen and Stangor 2001) adolescents evaluated straightforward rejection as morally reprehensible.

*TP 8: Yeah, it's just rude if they tease someone because they have Tourette's (Focus group 3)*

However, the majority of the participants differentiated their reciprocated relationships with other typically developing peers from the anticipated affiliations that would be formed with individuals with TS. A form of relationship was delineated which would be lacking in intimacy, companionship and closeness; three of the most important elements that define the quality of a friendship (Sullivan 2013):

PL3: *No, I wouldn't mind to hang out with a person with Tourette’s, but I would want my space as well. …I would want not every day hang out, not every day be together, not every day talk. Don’t want him to cling on to me. Yeah*

Moderator: *So, do you do that with your other friends?*

PL3: *Not really*

PL1: *So, he/she would be like a secondary friend?*

PL3: *He wouldn’t be at the same level, the same level as all my other friends*. (Focus group 1)

Participants would provide support for their peer with TS, assist them if necessary, advocate on their behalf if their rights were being threatened and defend them if they were disrespected. However, they would not actively socialise with them, thus describing a friendship that is based on benevolence and not on reciprocity. Their feelings therefore appeared to stem from a sense of duty and morality.

TP2: *I would hang around with them but not like everywhere. I would need my space to hang around with my other friends as well that are more like me. They wouldn’t be like my best friends, the one I call when things get bad like to help me. How could they? Right?* (Focus group 3)

Greater hesitancy towards engaging in relationships with individuals with TS became more apparent as the relationships deepened. As it was also acknowledged, in order for a romantic relationship to be formed, intimacy must be created between the two partners. However, in the case of individuals with TS participants claimed that closeness might be harder to achieve and pity, sympathy and a tendency towards over-solicitousness could be perceived as a potential barrier to genuine attraction.

TP 14: (on dating) *It depends on how comfortable I am around him, cause If I feel like guilty and sorry for him, want to help him then I don't think I can be in a relationship with him* (Focus group 4)

*PL3: I'm being honest yeah. I'm not being rude, I'd want to have somebody normal. I'm not saying they’re not normal* (individuals with TS). *But you know what I mean. I would mostly feel so sorry [for] him* (Focus group 1)

Adolescents perpetuated superficial relationships that were fuelled by a parenting attitude and the participants’ need to provide protection and social comfort to the disadvantaged ‘other’. Affiliations were perceived as a charitable and altruistic act that made the actor feel superior.

## Discussion

Previous studies which have examined typically developing youths’ attitudes towards individuals with TS have failed to explore the context of individual’s perceptions and the reasons why these views may develop. The current study compliments and expands upon the relevant literature by broadening our understanding about adolescents’ beliefs and perceptions about TS and their subsequent affective and behavioural reactions.

The grounded theory analysis provided an overarching theoretical framework for understanding the process by which individuals with TS are perceived by their typically developing peers. Misconceptions about TS which were mostly perpetuated by stereotypical media images prevailed among adolescents’ understanding about the condition. Participants who constructed TS as a disorder beyond the individuals control perceived their peers as being deprived of agency and strength and as straying from the boundaries of normalcy. The wide gap that was formed between ‘them’ and ‘us’ was accompanied by downwards social comparison (Finlay and Lyons 2000; Wills 1981). Individuals with TS were viewed as in need of assistance and a form of patronising help was offered. This kind of ‘benevolence stigma’ (Cohen and Struening 1962; Corrigan et al. 2001) in combination with the fear of being ‘socially contaminated’ inhibited the initiation of meaningful social relationships with individuals with TS. A close degree of contact was also impeded due to intergroup anxiety and a form of cognitive dissonance. On the other hand the limited number of participants that held individuals with TS responsible for their symptomology expressed a plenary desire for social distance. However, decidedly negative behavioural intentions were not solely limited to adolescents who elicited inferences of responsibility to people with TS indicating that attributional models of stigmatisation may be of secondary importance in the case of TS.

This theory suggests that it is likely that youth with TS would be peripheral to social networks in comparison to typically developing adolescents. Although the latter reported that peers with TS would mostly be socially accepted, the quality of the friendships that they would be willing to engage in would be different from the ones they would establish with their neurotypical peers. Previous research suggests that it is the quality of reciprocated friendships, rather than the number of relationships or the presence or absence of certain types of affiliations that can be associated with social adjustment and social skills, internalised distress and, ultimately, the quality of life of individuals with TS (Sullivan 2013). This study also highlights that stigma and discrimination do not necessarily manifest themselves in the form of overt antipathy and that prejudiced attitudes revealed can depend on the level of intimacy in question. As opposed to active rejection an individual with TS might endure a form of prejudice which can appear in the form of patronisation or neglect (Cuddy et al. 2007).

The imperative need for an educational component in an intervention for typically developing adolescents was exemplified by the participants’ skewed and inadequate knowledge about the condition. Adolescents’ curiosity about the condition, as indicated in the subtheme of ‘unsolicited questions’, further highlights their willingness to understand the core nature of TS. Most of the participants based their impressions of individuals with TS on various mediums of popular culture, verifying the notion that the media is a potent force that has a detrimental impact on the creation of perceptions (Mutz and Goldman 2010). Adolescents who reported having relatives with Tourette’s syndrome also endorsed commonly held stereotypes pertaining to the syndrome and acquired their knowledge from TV. In popular cultural portrayals the accuracy of symptomology has been wilfully abandoned and extreme cases of TS have prevailed over the typical ones. The participants’ stereotypical recollections of images of TS in the media are consistent with the findings of a recent study (Calder-Sprackman et al. 2014) which demonstrated that Tourette’s is used in films and television to exonerate the use of profanities and entertain the audience. Thus, for comic effect, Tourette’s syndrome has been presented as the ‘swearing disorder’ where as in fact although coprolalia can be encountered in individuals with TS, only a minority is afflicted. Indeed, the prevalence rate of coprolalia ranges from 10 to 30 % of the TS population and can be associated with severe symptomology (Eddy and Cavanna 2013; Freeman et al. 2009).

Participants placed the onus on the abnormalities that underpin the condition and the pronounced differences between ‘them’ and ‘us’. In anticipation of intergroup interaction participants alluded to the notion of intergroup anxiety. Consistent with previous research anxiety would be evoked by affective components, leading to discomfort (Stephan 2014). Uncertainty and unfamiliarity may accentuate negative emotions which in turn could lead individuals without TS to quickly disengage from intergroup interaction. Indeed, the current study suggests that uncertainty about how to react towards individuals that were perceived as different, individual’s self-doubt about their competence in facilitating interaction, and the expectancy that contact might be extremely difficult, exacerbated anxiety. The results of the study also suggest that psychological discomfort may stem from a form of cognitive dissonance. Individuals’ egalitarian values and positive evaluations towards their peers with TS conflicted with the identification of their peers with TS as outgroup members (Festinger 1962).

The adolescents in the study were also concerned about the negative social consequences interaction with an out-group member might entail and apprehensive about disapproval from other intragroup members. This would lead some to avoid and/or seek to create a distance between themselves and outgroup individuals (Stephan 2014). Indeed adolescents feared that a social relationship with a “blemished” individual (Goffman 1963) would erode their social standing. In line with the mechanism of evaluative conditioning, their own devaluation by their peers would merely be the result of being paired with a disliked stimulus; in this instance a peer with TS (Walther 2002). The phenomenon of the social contagion and spreading of stigma to individuals that do not possess the undesirable characteristic(s) is supported by much empirical evidence and acknowledged as a robust social phenomenon (Goffman 1963; Pryor et al. 2012). Studies suggest that the bearer of courtesy stigma does not necessarily have to have close and meaningful ties with a stigmatised person but the spill over effects could be a result of merely being observed in their presence (Hebl and Mannix 2003).

In an attempt to understand TS participants also evaluated the actor’s perceived responsibility in the manifestation of the condition. The vast majority of adolescents in the study attributed the causation of TS solely to factors that were beyond the individual’s control and therefore inferred reduced personal responsibility and decreased moral accountability. Simultaneously however the actor was viewed as a passive victim and TS as a deterministic and stable disorder that induced feelings of pity and sympathy towards the ‘sufferer’. The limited participants who perceived the cause of TS as high on personal control exhibited negative emotions towards their peers with TS. These findings are especially interesting when studied in conjunction with the attribution theory framework (Weiner 1986, 1995). According to Weiner’s attribution-emotion- action model the perceived level of responsibility and control an individual has over their actions may influence the observers’ emotional response towards them and their subsequent behaviour. More specifically low controllability may engender high levels of sympathy and pity and elicit positive behavioural intentions. Conversely, perceived controllability may evoke more negative emotions, high levels of anger and subsequently antisocial behavioural intentions. The current study suggests that in the context of TS the theory is only partially supported. Perceived uncontrollability over the tics determined the participants’ emotional reactions and the hypothetical peer elicited sympathy and pity. However, these emotions towards individuals with TS did not always increase the participants’ willingness to decrease social distance and did not necessarily elicit benign reactions. Thus, adolescents who attributed the causation of the condition to factors beyond the afflicted persons control still avoided a close degree of contact with the target individual, and pity did not translate to prosocial behaviour and a desire to help. Our findings highlight that attitudes and behavioural intentions towards adolescents with TS is a complicated matter associated with a variety of issues that do not solely relate to attributional models and blame. Therefore, attributing the causation of TS to factors beyond the inflicted person’s control cannot be perceived as a panacea for stigma reduction.

The findings also suggest that pity resulted from adolescents seeking out comparison with a group they viewed to be socially disadvantaged and wrongfully discriminated against. The theory of social comparison-based emotions maintains that affective reactions can be elicited by comparing oneself to superior others (upwards comparison) or inferior others (downwards comparison) (Smith 2000). In this instance a downwards comparison was activated that induced feelings of pity since individuals with TS were being perceived as members of a lower status group with limited prospects and life chances. Pity in the context of the study cultivated a form of benevolence stigma (Cohen and Struening 1962; Corrigan et al. 2001). Thus, people with TS were perceived as powerless, fragile, vulnerable; the disorder as a socially disabling condition to which people react with kindness but simultaneously akin to how parents treat their children. Consistent with the stereotype content model hypotheses the findings of this study suggest that pity results in a paternalistic form of prejudice (Cuddy et al. 2007). In contrast to direct and aggressive forms of discrimination patronisation is masked by a seemingly positive behaviour towards the recipient. However, an unequal power relation between the ‘sufferer’ and the one who pities them was implied. This entails a subordination and an unseemly dependency. The benevolent and ablest helper provides assistance to the less fortunate and expresses affection but not respect. The latter are viewed as in need of help and are construed as unable to manage on their own without outside intervention. The asymmetric power relationship that has been formed implies the superiority of the patron and the inferiority of the receiver. Indeed, as Nietzsche has pointed out actions taken because of pity are not necessarily altruistic (Nussbaum 1994). Rather they mostly stem from a selfish need to eliminate the internal pain caused by viewing the suffering of another. This form of paternalistic prejudice or benevolence stigma is more subtle since it manifests in the form of protective tendencies although concealing condescension. It is also considered as positive behaviour from the point of the perceiver and therefore more likely to remain unchallenged. Indeed in contrast to overt discrimination which was perceived by the study participants as moral transgression, individuals maintained that they would behave in an ostensibly unprejudiced manner towards the out-group members. Thus, providing patronising help was conceived as motivated by moral principles and values and therefore justified as benevolent. Therefore, in keeping with previous research the findings of this study suggest that people try to behave in a manner that appears to be moral and maintain a positive social identity through exhibiting what is believed to be positive behaviour towards out-groups (Ellemers et al. 2013). Nevertheless, Fominaya et al. (2016) suggest that being viewed as an object of pity can have detrimental impacts on a person’s sense of worth, self-esteem and sense of empowerment.

Pity and benevolent attitudes also aroused a strong desire for social distance among adolescents and in this sense, our findings are consistent with those of Fominaya et al. (2016) who demonstrate that pity regarding mental illness is correlated to decreased anger but also increased social avoidance. It appears therefore that one’s desire to provide help may be impended by the desire to maintain a social distance. Indeed, in line with the Behaviours from Intergroup Affect and Stereotypes (BIAS) map (Cuddy et al. 2007) pity may engender an active facilitation, in this instance patronisation, but also a passive form of harm which is presented in the form of neglect and avoidance. Participants wanted to avoid the negative feelings that pity evokes and maintain distance from the unfortunate other or help him/her without wanting to establish a reciprocal relationship.

### Limitations

While this research makes important strides in understanding how individuals with TS are perceived by their peers, the findings should be interpreted within the context of the current study design. First, the study was limited by a small and homogeneous sample size, and the use of one data collection site. The participants were recruited through convenience sampling making the findings of the study specific to a particular school and target group and therefore difficult to generalise to the larger population of adolescents in the United Kingdom. Furthermore, although the school in question is characterised by ethnic diversity, due to the stringent participants’ selection criteria, the sample of individuals that were from an ethnic background other than White-English were underrepresented. Convenience sampling also led to unequal representation of males (only 23 % of the sample) which suggests that the results may be limited when extrapolating both genders. A variety of studies suggest that females exhibit more positive attitudes towards individuals with disabilities and therefore our findings may be positively skewed (Goreczny et al. 2011).

Other limitations relate to the stimulus presented to the participants. In the video the young woman with TS exhibited tics that were both high in frequency, intensity and severity which may have negatively influenced the participants’ willingness to interact with individuals with Tourette’s syndrome (Woods et al. 1999). Furthermore, Boudjouk et al. (2000) demonstrates that adolescent females with tics are evaluated less positively than males with tics. Finally, by viewing segments of a video as opposed to an adolescent with TS in vivo the external validity of the study is limited. Therefore, hesitancy voiced by participants may have been due to a lack of familiarity rather than TS itself.

Further methodological limitations emerged as a result of using focus groups. In at least two focus groups a “collective” voice emerged that mostly represented the most influential participants of the group (Kitzinger 1994). Consensus within the group and adolescents’ need for peer approval may have limited some participants’ willingness to express diverse views or opinions that could have been considered less socially desirable (Sullivan 2013). More specifically, a number of the study females exhibited a tendency to conform to the opinions of more confident and dominant peers and to rephrase and adjust their answers in order for them to comply with social desirable responses. Potential response bias could have also been due to the presence of a staff member in the room where the focus groups were taking place. Although the ‘authority figure’ did not participate in any of the discussions and was sitting silently in the back of the room, her mere presence may have inhibited the participants from expressing less socially desirable responses. Furthermore, despite the researcher’s effort to get all the participants involved and to encourage group building, not all adolescents participated equally. Thus, some responses were no longer than a few words and the participants were not able to fully engage in the conversations and the structure of the focus groups were more akin to an interview.

Finally, social desirability bias may provide a plausible explanation as to why the participants attributed the act of social exclusion solely to other peers and not to themselves. Driven by self-presentation and prejudice concerns participants may have underrepresented behaviours that they considered to be socially inappropriate. Overrepresentation of one’s own kindness and generosity may also be due to the “holier than thou” effect; people tend to perceive themselves as uniquely and unrealistically moral, ethical and altruistic in comparison to their peers (Epley and Dunning 2000).

### Implications for Practice and Future Directions

Despite the identified limitations the study builds on scarce literature on adolescents and TS and advances knowledge about the barriers to social inclusion for individuals with TS. Targeting and addressing specific misconceptions in an educational intervention may be important because as studies suggest erroneous beliefs may be harder to change and may adversely affect the understanding of new information (Taylor and Kowalski 2004). The findings of this study will help enrich educational/ anti-stigma interventions on the transmission of knowledge. The study highlights that the media is the primary source of information for adolescents that is used to inform about TS and therefore can potentially be used for educational purposes. Indeed, future interventions addressed towards adolescents should utilise and exploit its dynamic role.

The findings also highlight that the stereotype of a distressed and disempowered individual with TS should be demythologised. Within this context the emphasis should be placed on the contextual factors that impact the biological processes rather than solely attributing tics to defective genes. By presenting the antecedents that facilitate TS expression people with TS can be viewed as less distinct (Conelea and Woods 2008). The nature of control over one’s tics should also be addressed. Thus, the ability for one to suppress the tics even for a prolonged period of time may attenuate unpredictability and ultimately make individuals with TS appear less dangerous and disempowered by the condition. Conversely, emphasis on a biogenetic model and lack of any control over the symptomology can encourage the notion of socially distinct and even ‘dangerous’ and subsequently widen the gap between ‘them’ and ‘us’.

Future interventions also need to augment adolescents’ willingness to help individuals with TS but without perpetuating the image of helplessness. Recent studies suggest that helping behaviour is not necessarily contingent to stereotypical depictions that elicit pity (Kamenetsky et al. 2016). Social inclusion can be achieved by communicating images of individuals with TS that are more congruent with typically developing peers’ self-image. Empathy can be cultivated by addressing and focusing on the similarities between the stigmatised and the norms and not ruminating on deficits and differences (Campbell et al. 2004).
